# Testing EEG functional connectivity between sensorimotor and face processing visual regions in individuals with congenital facial palsy

**DOI:** 10.3389/fnsys.2023.1123221

**Published:** 2023-05-05

**Authors:** Thomas Quettier, Antonio Maffei, Filippo Gambarota, Pier Francesco Ferrari, Paola Sessa

**Affiliations:** ^1^Department of Developmental and Social Psychology, University of Padua, Padua, Italy; ^2^Padova Neuroscience Center (PNC), University of Padua, Padua, Italy; ^3^Institut des Sciences Cognitives Marc Jeannerod, CNRS/Université Claude Bernard Lyon 1, Bron, France

**Keywords:** Moebius syndrome, facial palsy, facial expressions, motor simulation, EEG functional connectivity, face processing

## Abstract

Moebius syndrome (MBS) is characterized by the congenital absence or underdevelopment of cranial nerves VII and VI, leading to facial palsy and impaired lateral eye movements. As a result, MBS individuals cannot produce facial expressions and did not develop motor programs for facial expressions. In the latest model of sensorimotor simulation, an iterative communication between somatosensory, motor/premotor cortices, and visual regions has been proposed, which should allow more efficient discriminations among subtle facial expressions. Accordingly, individuals with congenital facial motor disability, specifically with MBS, should exhibit atypical communication within this network. Here, we aimed to test this facet of the sensorimotor simulation models. We estimated the functional connectivity between the visual cortices for face processing and the sensorimotor cortices in healthy and MBS individuals. To this aim, we studied the strength of beta band functional connectivity between these two systems using high-density EEG, combined with a change detection task with facial expressions (and a control condition involving non-face stimuli). The results supported our hypothesis such that when discriminating subtle facial expressions, participants affected by congenital facial palsy (compared to healthy controls) showed reduced connectivity strength between sensorimotor regions and visual regions for face processing. This effect was absent for the condition with non-face stimuli. These findings support sensorimotor simulation models and the communication between sensorimotor and visual areas during subtle facial expression processing.

## Introduction

Moebius Syndrome (MBS; Moebius, 1888) is a rare congenital neurological disorder characterized by the affection of cranial nerves VI and VII (Briegel, 2006), leading to impaired lateral eye movements and complete or nearly complete–usually bilateral–facial paralysis. In addition, other congenital conditions are sometimes present, such as limb anomalies (e.g., clubfoot and missing/underdeveloped fingers or hands; Richards, 1953; Verzijl et al., 2003). On the psychological side, individuals with MBS show difficulties in social interactions with different degrees of severity, mostly because they cannot express their emotions to others through their faces (Bogart and Matsumoto, 2010). Therefore, *per* the definition, MBS individuals are characterized by a deficit in the *production* of facial expressions.

A prominent theoretical model supports the existence of a close relationship between production (e.g., of gestures and facial expressions) and *perception* (e.g., of gestures and facial expressions) (Preston and de Waal, 2002; Rizzolatti and Sinigaglia, 2016). Several studies provided evidence in favor of shared neural representations of emotional facial expressions between production and perception in different brain regions. These include the inferior, middle, and superior frontal gyri, the amygdala, and the insula (Molenberghs et al., 2012), suggesting that these shared representations could hinge on mirror mechanisms (Van Overwalle and Baetens, 2009). Overall, this “shared representational system” is thought to subserve others’ social understanding and emotion perception by *motor simulation* (Goldman and Sripada, 2005; Bastiaansen et al., 2009; Likowski et al., 2012). It has recently been hypothesized that the primary mechanism through which motor simulation supports emotion perception is that of an iterative communication between motor, premotor, somatosensory cortices (overall the sensorimotor system), and the visual cortices (Wood et al., 2016a,b). Specifically, iterative communication would increase the quality/precision of the visual percept, allowing for more efficient discriminations of facial expressions (Wood et al., 2016a,b). In this context, *facial mimicry*, the visible or invisible contraction of the facial muscles congruent with the observed expression, is conceived as a peripheral manifestation of the central sensorimotor simulation. Sensorimotor simulation models (Goldman and Sripada, 2005; Bastiaansen et al., 2009; Likowski et al., 2012; Wood et al., 2016a,b) assume that facial mimicry contributes to the motor simulation through the feedback provided to motor areas.

Within this theoretical framework, MBS individuals should be characterized by altered facial feedback to the central nervous system (especially to the motor cortex) because of facial palsy, and, as a consequence of the congenital condition, they should not have (at least complete) facial motor programs for facial expressions. In short, MBS individuals could not efficiently exert the hypothesized sensorimotor simulation mechanism in recognizing/discriminating facial expressions. Nevertheless, it is possible that by mechanisms of plasticity and compensation, individuals with MBS can achieve normotypical performances (Vannuscorps et al., 2020) and have developed alternative and efficient neural pathways for the recognition/discrimination of facial expressions (Sessa et al., 2022). Therefore investigations using neuroimaging techniques are necessary to explore the neural bases of the emotional expression processing in MBS individuals beyond their behavioral performance (in terms of accuracy and/or reaction times) that could be normotypical.

Due to the absence of a shared representational system/motor simulation, one might expect that the neurological population of MBS is characterized by: (a) impaired recognition/discrimination of emotional facial expressions (in the case of lack of compensation) and (b) lower degree of connectivity (compared to healthy individuals) between sensorimotor and visual systems during subtle discrimination of emotional facial expressions.

In a previous investigation, our findings corroborated the hypothesis of compensatory mechanisms, which, in terms of neural pathways, might hinge on the recruitment of different brain regions in MBS compared to healthy individuals during emotional expression discrimination tasks (Sessa et al., 2022). The specific aim of the present study, instead, is precisely to test the predicted reduced connectivity *between sensorimotor and visual systems in MBS, compared to healthy controls*.

To this aim, we administered our participants, healthy and MBS, an emotional expression discrimination task. Cortical activity was recorded with high-density electroencephalography (hd-EEG) to investigate functional connectivity, i.e., the strength to which activity between a pair of brain regions covaries or correlates over time (Lachaux et al., 1999). In the case of EEG signals, phase synchronization is one of the most widely used indexes to investigate functional connectivity under the assumption that the phase of two oscillations of different brain regions should be correlated if the two regions are functionally connected (Lachaux et al., 1999; López et al., 2014). We computed the phase locking value (corrected imaginary phase locking value; ciPLV; see section “Materials and methods”) of the beta oscillatory activity according to the previous and convincing evidence that links the processing of stimuli with affective value to long-distance EEG connectivity in the beta band (Aftanas et al., 2002; Miskovic and Schmidt, 2010; Zhang et al., 2013; Wang et al., 2014; Kheirkhah et al., 2020; Kim et al., 2021).

Although not made explicit by the motor simulation models (Wood et al., 2016a,b), the visual cortices involved in the iterative communication must primarily entail regions delegated to the visual analysis of faces. The most accredited neural model of face processing, i.e., the distributed model of face processing by Haxby et al. (2000) and Haxby and Gobbini (2011), encompasses, indeed, a *core system* for faces’ visual processing (comprising the fusiform face area, the occipital face area, and the posterior superior temporal sulcus; Haxby et al., 2000; Grill-Spector et al., 2004; Winston et al., 2004; Yovel and Kanwisher, 2004; Ishai et al., 2005; Rotshtein et al., 2005; Lee et al., 2010; Gobbini et al., 2011), and an *extended system* for additional non-visual processing steps, including the attribution of meaning to facial expressions in terms of emotion (comprising the sensorimotor cortices; Haxby and Gobbini, 2011).

Based on this knowledge, we expected *phase synchronization* (i.e., the connectivity index) *between the sensorimotor system and the core system* to be significantly greater in healthy participants than in MBS participants. As preliminary evidence to circumscribe and characterize this effect as face-sensitive, we included an identical task but involving non-face stimuli (i.e., animal shapes). We did not expect to observe any difference between healthy participants and MBS participants with regard to the strength of the connectivity index for non-face stimuli.

## Materials and methods

### Participants and task

In this research we enrolled 14 adults, seven MBS participants (MBS group: MBS 4 females and 3 males, mean age = 40, 43 years; s.d. = 11,03) and seven healthy control participants. Controls were matched for age, gender and level of education. Participants in the MBS group had a diagnosis of unilateral or bilateral facial paralysis (Terzis and Noah, 2003). See Table 1 for demographic data and clinical information for MBS participants. All the participants did not report any psychiatric or physical illness.

**TABLE 1:** Demographic data and clinical information for MBS participants.

Participant	Age	Gender	Cranial nerves involved	Disfunction
MBS1	54	Male	Abducens Nerve (VI)	No lateral eye movements
			Facial Nerve (VII)	Facial palsy
MBS2	57	Males	Abducens Nerve (VI)	No lateral eye movements
			Facial Nerve (VII)	Facial palsy
MBS3	38	Male	Abducens Nerve (VI)	No lateral eye movements
			Facial Nerve (VII)	Facial palsy
MBS4	25	Female	Abducens Nerve (VI)	No lateral eye movements
			Facial Nerve (VII)	Facial palsy
MBS5	65	Female	Facial Nerve (VII)	Facial palsy
MBS6	39	Female	Abducens Nerve (VI)	No lateral eye movements
			Facial Nerve (VII)	Facial palsy
MBS7	34	Female	Abducens Nerve (VI)	No lateral eye movements
			Facial Nerve (VII)	Facial palsy

**FIGURE 1:**
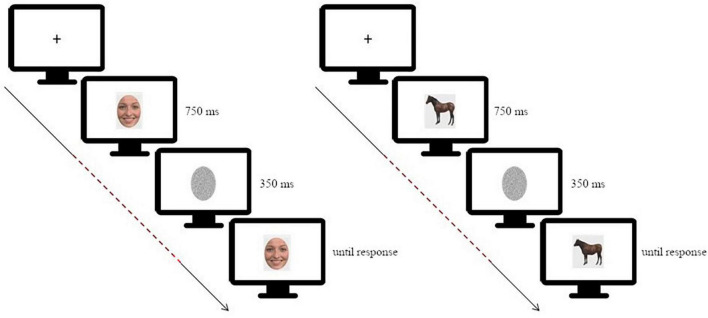
Schematic depiction of the experimental paradigm [on the **left**: for the facial expression discrimination task (morphing continua happiness-disgust); on the **right**: for the animal shape discrimination task]. The dashed line highlights the time window considered for the connectivity analysis, starting at target stimulus onset and ending before test stimulus onset. Adapted from Sessa et al. (2022).

Participants performed a simple change detection task in which they had to judge if a test image was different or not compared to a target image. This task has been successfully used to investigate changes in neural activity, as well as connectivity, during face processing (Wood et al., 2016a; Lomoriello et al., 2021; Maffei and Sessa, 2021a). In each trial the target image was presented on a screen for 750 ms, masked with noise for 350 ms and then followed by the test image which lasted on screen until response (Figure 1).

The stimuli were 11 digital images of faces and animals.

**FIGURE 2:**
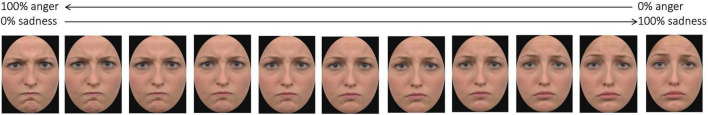
Example of stimuli of one of the morphing continua. Adapted from Sessa et al. (2022).

For each category, we created the morphing continuum as follows: for the face stimuli we had two continua, one ranging between the expression of anger and the expression of sadness, and one ranging between the expression of happiness and the expression of disgust (Figure 2 shows stimuli of one of the morphing continuum); for the animal stimuli the continuum ranged between the image of a horse and the image of a cow, both presented in the same posture. Each continuum started with an expression/animal shape consisting in 100% of one expression/animal shape and 0% of the other (i.e., 100% anger–0% sadness or 100% happiness–0% disgust; 100% horse–0% cow), and then changed in 20% increments/decrements until reaching the opposite end (e.g., 0% sadness–100% anger, 0% horse–100% cow). The stimuli are available at the following link of the Open Science Framework repository: osf.io/krpfb.

On each trial, the *target* stimulus was randomly selected from one of the continua, then it was followed by the mask, and finally the *test* stimulus was presented. This latter test stimulus was selected from the same continuum of the target pseudorandomly, such that it was maximum 40% apart on the morph continuum, to control for discrimination difficulty across participants.

### EEG acquisition and pre-processing

Electroencephalography activity was recorded from 128 channels using an HydroCel Geodesic Sensor Net (HCGSN-128) connected to a Geodesic EEG System (EGI GES 300). Data were collected continuously with a sampling rate of 500 Hz using the vertex as online reference. Channel impedance was kept under 60 kΩ. For the purpose of the present research we analyzed the preprocessed data used in Sessa et al. (2022). Briefly, pre-processing consisted in downsampling the data to 250 Hz and band pass filtering (0.1–45 Hz), epoching between −500 to 1500 ms relative to target onset, rejection of artifactual components after ICA using the ICLabel algorithm (Pion-Tonachini et al., 2019), bad channel interpolation and referencing to the average of all channels. Further details regarding the pre-processing can be found in Sessa et al. (2022). The preprocessed data as well as the pre-processing script can be accessed at https://osf.io/krpfb/.

In order to estimate brain activity from the preprocessed scalp recordings, we first created a forward model using the three-layer boundary element method (BEM) from OpenMEEG, implemented in Brainstorm, and then estimated an inverse solution with the weighted Minimum Norm Estimation (wMNE) with default parameter. Finally, the estimated distributed source activity was downsampled to the 148 cortical parcels of the Destrieux et al. (2010), averaging the activity of all the vertices included in each parcel.

### Functional connectivity analysis

Functional connectivity was estimated using the phase locking value, which is a widely used statistic able to quantify the degree of phase synchronization in a given frequency band (Lachaux et al., 1999; Maffei and Sessa, 2021a,b). Specifically, we employed an updated version of the original PLV statistics, recently introduced by Bruña et al. (2018): the corrected imaginary part of PLV (ciPLV). As with the original PLV, ciPLV analysis first requires a time-frequency decomposition of the signals, which can be obtained either through wavelets or applying the Hilbert transform on narrow-band filtered signals. Then, for each pair of signals, ciPLV is estimated as the imaginary part of the phase difference between the two signals. Contrary to the classic PLV, taking only the imaginary part of the phase difference allows to discard any zero-lag interactions, making ciPLV robust to volume conduction and/or source leakage which are known to inflate classic PLV (Bruña et al., 2018).

**FIGURE 3:**
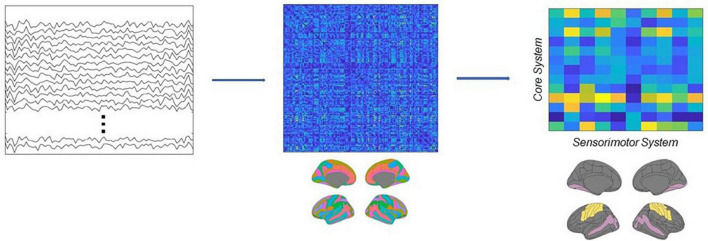
Schematic depiction of the analytical pipeline. Source activity was reconstructed from EEG recordings, then connectivity was estimated using the ciPLV in the beta band (13–30 Hz). Finally, the connectivity strength between the core and the sensorimotor systems was extracted from the full adjacency matrices.

In this research, we first band-pass filtered the source estimated activity in the beta range (13–30 Hz), then applied the Hilbert transform to derive the analytical representation of the signals, and finally computed ciPLV for each pair of ROI of the Destrieux atlas in the time range between the onset of the target image and the onset of the test image (0–1100 ms). This workflow resulted in a 148 × 148 symmetric matrix *M*, where each entry represents the connectivity strength between each pair of regions. Then we subsampled this matrix, in order to extract a new rectangular matrix *R*, where the columns identify the regions belonging to the core system of the face processing network (Haxby and Gobbini, 2011; Maffei and Sessa, 2021a) and the rows identify the primary and secondary motor and somatosensory cortices (see Supplementary material). Each entry of this matrix thus represents the value of connectivity between a ROI belonging to the *core system* and a ROI belonging to the *sensorimotor system*. Finally, we computed the connectivity strength between the two systems as the sum of the matrix, *w* = ∑_*i*,*j*_ *R*_*i*,*j*_ see Figure 3.

### Statistical analysis

The main goal of this research was to test if participants with MBS are characterized by an impaired connectivity between visual and somatomotor regions during the processing of facial expressions. To test this hypothesis we performed an independent samples *t*-test on the connectivity strength estimated from trials in which participants were presented with facial expressions. The null hypothesis was that the two groups, MBS and controls, should not differ in the degree of functional connectivity between visual and sensorimotor regions. We also performed an additional analysis on the connectivity strength estimated from trials in which participants were presented with images of animals as a preliminary assessment to test the face-sensitivity of this effect. We present this analysis with caution as we are aware of the limitations of the statistical approach due to the extreme rarity of the MBS condition.

For the readers interested instead in analysis of the behavioral performance we refer to Sessa et al. (2022).

## Results

**FIGURE 4:**
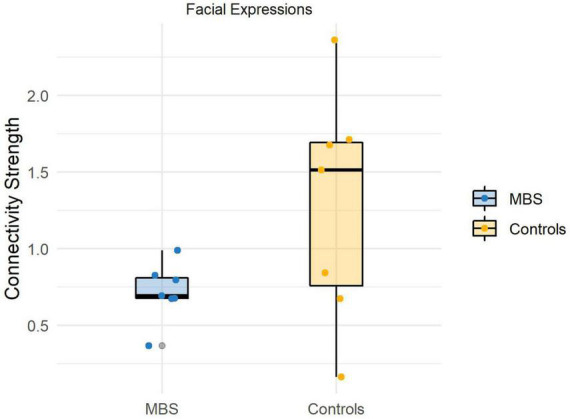
Individual connectivity strengths during facial expressions processing in the beta band (13–30 Hz) for each group.

Before running the statistical comparisons, we used the Shapiro-Wilk statistics to check for the normality of the data, and the test suggests that there is no violation of normality (W_face_Moebius_ = 0.92, *p* = 0.52; W_face_Controls_ = 0.96, *p* = 0.83;W_animal_Moebius_ = 0.93, *p* = 0.61; W_animal_Controls_ = 0.97, *p* = 0.93). The analysis performed for the *Face* condition revealed a significant difference [t_(12)_ = −1.91, *p* < 0.05, *d* = −1.2] between the two groups, showing that participants affected by facial palsy, compared to healthy controls, were characterized by a reduced connectivity strength between sensorimotor regions and visual regions comprised in the core system of the face processing network (M_MBS_ = 0.7, M_CTRL_ = 1.2; see Figure 4). Conversely, the analysis performed for the *Animal* condition did not reveal any significant difference between the two groups [t_(12)_ = −1.36, *p* = 0.1].

## Discussion

Over the last 20 years, various models of (sensori)motor simulation (Goldman and Sripada, 2005; Bastiaansen et al., 2009; Likowski et al., 2012; Wood et al., 2016a,b) have been proposed, all sharing the central theoretical hypothesis that facial expressions’ recognition and fine discrimination are supported by the recruitment–in the observer–of motor programs congruent with the facial expression observed, positing a relationship between production and recognition abilities. Therefore, it follows that individuals affected by clinical conditions limiting the production abilities, should also show recognition deficits. Although the congenital disorder in MBS subjects could trigger plastic cerebral modifications leading to alternative and efficient neural pathways to recognize emotional expressions, the deficiency of the simulation mechanism–if true–should necessarily translate into reduced functional connectivity between sensorimotor and visual systems, which is a central tenet of the most recent sensorimotor simulation models (see Wood et al., 2016b).

Here, we wanted to test this hypothesis by comparing the functional connectivity between the core system and the primary and secondary motor and somatosensory cortices in MBS and healthy individuals. We implemented a change detection task of facial expressions and animal shapes, and studied brain connectivity in terms of phase locking (corrected imaginary phase locking value; ciPLV; see section “Materials and method”). We restricted our analysis to beta oscillatory activity as the best approach to capture putative long-distance EEG connectivity involved in processing stimuli with affective value (Aftanas et al., 2002; Miskovic and Schmidt, 2010; Wang et al., 2014; Zhang et al., 2013; Kheirkhah et al., 2020; Kim et al., 2021).

The results supported our hypothesis. Indeed, as expected, for the facial expressions discrimination, reduced connectivity strength between sensorimotor regions and visual regions comprised in the core system of the face processing network was found in participants affected by facial palsy when compared to the matched healthy controls. Such a difference in the connectivity strength between the two groups was not observed for the animal shape condition. We are aware that the rarity of the syndrome and, consequently, the magnitude of the sample size cannot allow us to (statistically) conclude that the effect is selective for facial expressions.

Nevertheless, this reduction in the connectivity index for facial expressions was significant and, as such, it might indicate that the simulation mechanism is (at least) deficient in individuals with MBS. This result perfectly aligns with our hypothesis, since the alteration or absence of cranial nerves VI and VII as a consequence of the congenital condition restricts facial feedback to the central nervous system, and, more importantly, impairs the development (at least complete) of facial motor programs for facial expressions. Consequently, MBS individuals should not exhibit connectivity between the sensorimotor and the visual systems in the absence of a simulation mechanism (or, in the case of residual muscular functioning, they should exhibit reduced connectivity when compared to healthy individuals).

If the functional significance of this connectivity, as predicted in the model by Wood et al. (2016b), is to favor the fine processing of emotional facial expressions, one might expect that subjects with MBS should be less efficient in those tasks that require this type of processing. To note, however, studies that have investigated the ability to recognize emotional expressions in MBS individuals have produced conflicting results (e.g., De Stefani et al., 2019 for a review), although the most convincing evidence seems to indicate that the individuals with the syndrome may exhibit normotypical performance, at least in terms of correctness (i.e., accuracy) when recognizing/discriminating emotional faces (Rives Bogart and Matsumoto, 2010; Vannuscorps et al., 2020; Sessa et al., 2022). These last results, on the other hand, are in line with compensatory/plasticity mechanisms, plausibly starting from birth, as recently supported by a recent study by Sessa et al. (2022). This last study, indeed, provided evidence in favor of the recruitment of an alternative neural pathway in Moebius individuals (vs. healthy controls), which does not seem to involve the motor and somatosensory regions, but rather more ventral areas (from the occipital face area/fusiform face area to the anterior temporal lobe; compatible with the proposals by Duchaine and Yovel, 2015; Pitcher and Ungerleider, 2021) that in healthy individuals contribute to the processing of emotional expressions although preferentially involved in the processing of form information, for instance, for face identity processing (Vuilleumier et al., 2001; Ishai et al., 2004; Ganel et al., 2005; Xu and Biederman, 2010).

The present study has some limitations which should be mentioned.

First, the level and extension of the nerves alteration in MBS are different from one patient to another patient. From this point of view and due to the extreme rarity of the syndrome, it is not always possible recruiting a homogeneous group, so that one patient out of seven patients had a deficit of the facial/VII nerve alone in the absence of a concomitant impairment of the abducens/VI nerve (see Table 1).

Second, another potential limitation regards the smile surgery that allows MBS individuals to produce smile-like facial movements. Crucially, after smile surgery, MBS could develop smile-like motor programs and the associated motor representation, which could be also potentially dysfunctional for simulation. In the present study, the experimental procedure also envisaged the fine discrimination of facial expressions of happiness. However, the behavioral and connectivity analyses were not carried out on each category of emotional expression separately. As a consequence we cannot examine whether the processing (at the behavioral and neural level) of the expressions of happiness in patients who received smile surgery (all but one in our sample) differ from other emotional expressions.

Third, in the present study, we have used static facial expressions to investigate the impact of congenital facial palsy on the connectivity between the sensorimotor and visual systems. However, it is important to note that the dorsal pathway, which is involved in the processing of facial expressions, is more sensitive and more strongly recruited when dynamic rather than static facial expressions are processed (Duchaine and Yovel, 2015). This suggests that the sensorimotor simulation mechanism is more strongly triggered by dynamic facial expressions. Therefore, in the present study, we might have underestimated the impact of congenital facial palsy on the connectivity between the sensorimotor and visual systems. Future studies should consider using dynamic facial expressions to provide a more accurate understanding of the impact of congenital facial palsy on the sensorimotor and visual systems.

To conclude, our results support sensorimotor simulation models and the communication between sensorimotor and visual regions of the core system during subtle facial expression discrimination. Furthermore, they indicate that this communication is atypical in MBS individuals for facial expression processing.

## Data availability statement

Publicly available datasets were analyzed in this study. This data can be found here: https://osf.io/krpfb/.

## Ethics statement

The studies involving human participants were reviewed and approved by the Ethics Committee of the University of Padua (Protocol No. 2855). The patients/participants provided their written informed consent to participate in this study.

## Author contributions

TQ: methodology and writing—original draft. AM: conceptualization, methodology, formal analysis, and writing—review and editing. FG: methodology and writing—review and editing. PF: conceptualization and writing—review and editing. PS: conceptualization, supervision, project administration, funding acquisition, and writing—review and editing. All authors contributed to the article and approved the submitted version.
